# Supplementary material for: The Incidence, Aetiology and Clinical Course of Serious Infections Complicating Biological and Targeted Synthetic Disease-Modifying Antirheumatic Drug Therapy in Patients with Rheumatoid Arthritis in Tropical Australia
Source: Pathogens. 2024 Oct 29;13(11):943. doi: 10.3390/pathogens13110943 (PMC11597851; doi:10.3390/pathogens13110943)

Supplementary Table S1. The association between the baseline demographic and clinical characteristics of the patients and the development of serious infection while receiving biologic therapy.

	**All who developed a serious infection n=74**	**Single serious infection n=50**	**Multiple serious infections n=24**	**p**
Age at first infection	61 (56-69)	61 (56-64)	65 (57-71)	0.25
Female Gender	56 (76%)	38 (76%)	18 (75%)	1.00
**First Nations Australian**	**12 (16%)**	**5 (10%)**	**7 (29%)**	**0.048**
Urban address	39 (53%)	28 (56%)	11 (46%)	0.41
Remote address	18 (24%)	11 (22%)	7 (29%)	0.57
**Charlson Comorbidity Index**	**2 (1-3)**	**2 (1-3)**	**3 (2-4)**	**0.001**
Severe Comorbidity (CCI ≥5)	7 (9%)	3 (6%)	4 (17%)	0.20
**Cardiovascular disease**	**12 (16%)**	**3 (6%)**	**9 (38%)**	**0.001**
**Lung disease**	**20 (27%)**	**9 (18%)**	**11 (46%)**	**0.02**
Diabetes	6 (8%)	4 (8%)	2 (8%)	1.0
Renal disease	1 (1%)	0	1 (4%)	0.32
Liver disease	1 (1%)	0	1 (4%)	0.32
Time on biological therapy (years)	5 (2-10)	5 (2-9)	6 (3-10)	0.86
**Seropositive rheumatoid arthritis**	**51/66 (77%)**	**33/47 (70%)**	**18/19 (95%)**	**0.049**
**Joint count**	**26 (22-31)**	**25 (20-29)**	**30 (27-36)**	**0.001**
**Corticosteroids at baseline**	**20/64 (31%)**	**18/44 (41%)**	**2/20 (10%)**	**0.02**
TNFi ever	67 (91%)	44 (88%)	23 (96%)	0.42
Adalimumab ever	42 (57%)	29 (58%)	13 (54%)	0.76
Golimumab ever	13 (18%)	8 (16%)	5 (21%)	0.75
Etanercept ever	27 (36%)	15 (30%)	12 (50%)	0.09
Certolizumab ever	8 (11%)	4 (8%)	4 (17%)	0.42
Infliximab ever	5 (7%)	2 (4%)	3 (13%)	0.32
JAKi ever	3 (4%)	2 (4%)	1 (4%)	1.0
Baricitinib ever	0	0	0	-
Tofacitinib ever	3 (4%)	2 (4%)	1 (4%)	1.0
Abatacept ever	10 (14%)	7 (14%)	3 (13%)	1.0
Tocilizumab ever	30 (41%)	20 (40%)	10 (42%)	0.89
Rituximab ever	22 (30%)	15 (30%)	7 (29%)	1.0

Numbers represent the median (interquartile range) or absolute number (%)
CCI: Charlson Comorbidity Index; TNFi: Tumour necrosis factor inhibitor; JAKi: Janus kinase inhibitor

Supplementary Figure S1. CONSORT diagram of identification for patients for the study

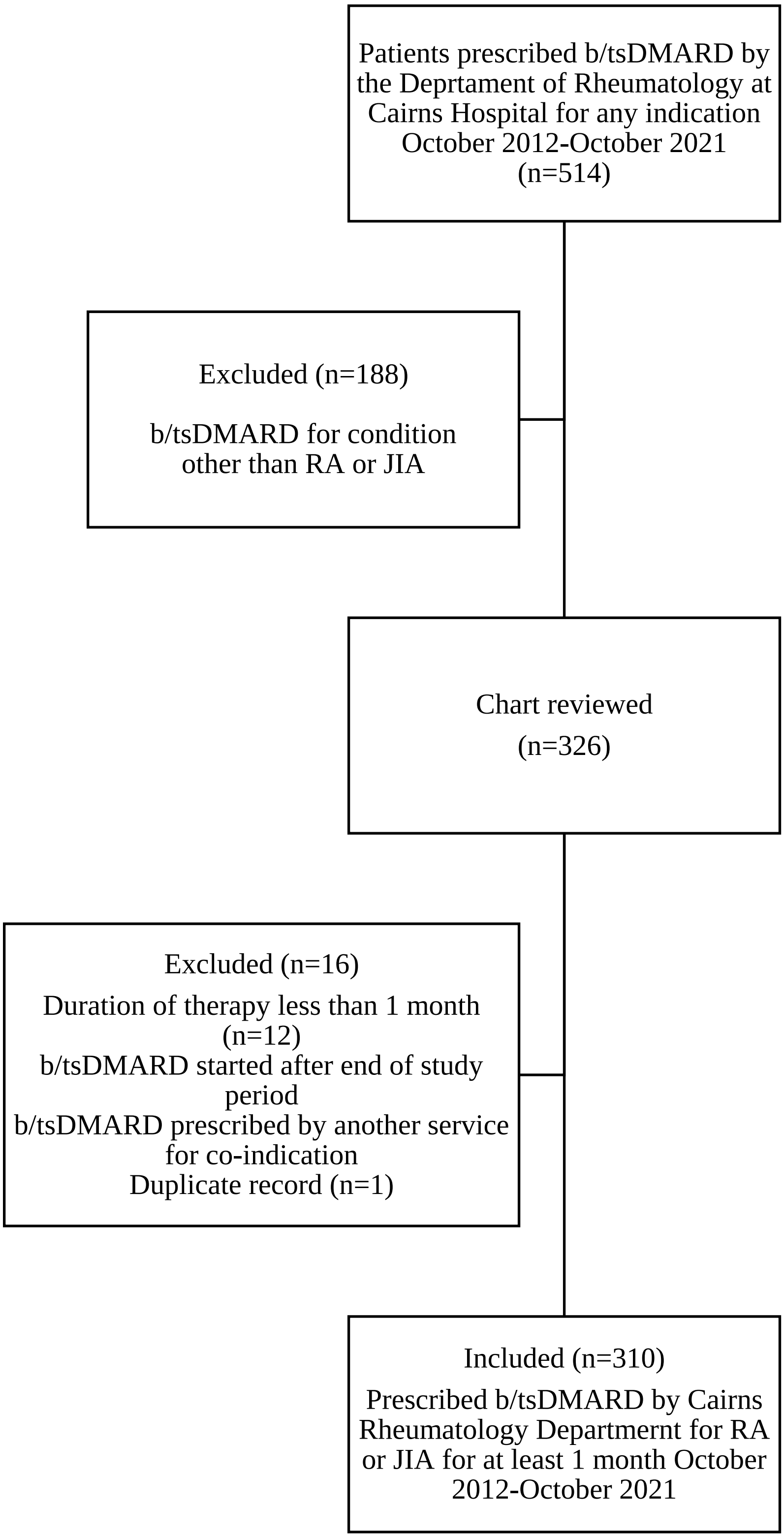

RA: rheumatoid arthritis
JIA: juvenile idiopathic arthritis

Supplementary Figure S2. Proportions of individuals with A. accessible chest x-ray results and B. accessible immunological tests for latent tuberculosis infection, stratified by site of initiation of b/tsDMARD therapy.

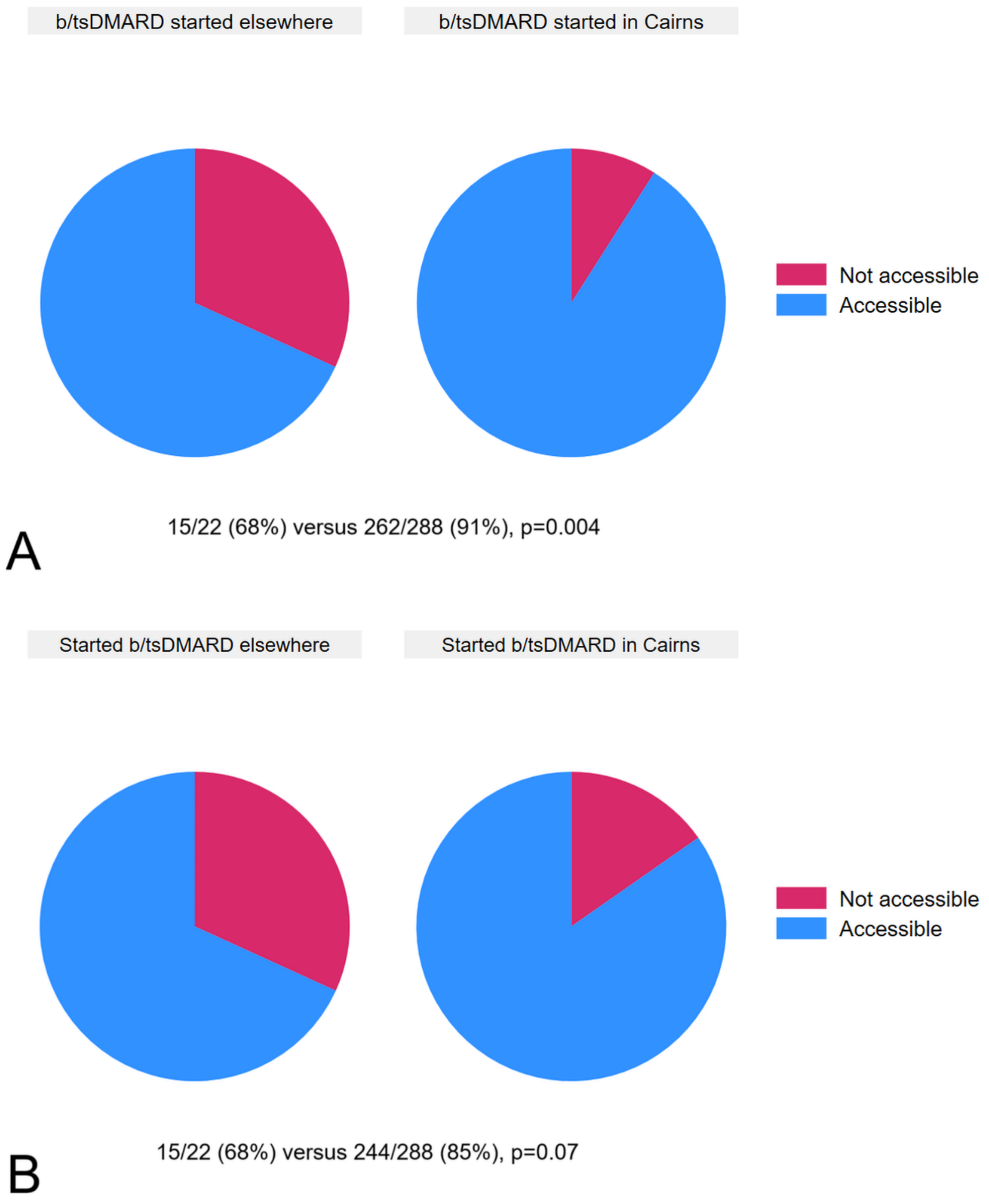

Supplementary Figure S3. Trends in ability to access A. chest x-ray test results and B. immunological screening tests for tuberculosis in patients initiating b/tsDMARD therapy at Cairns Hospital.

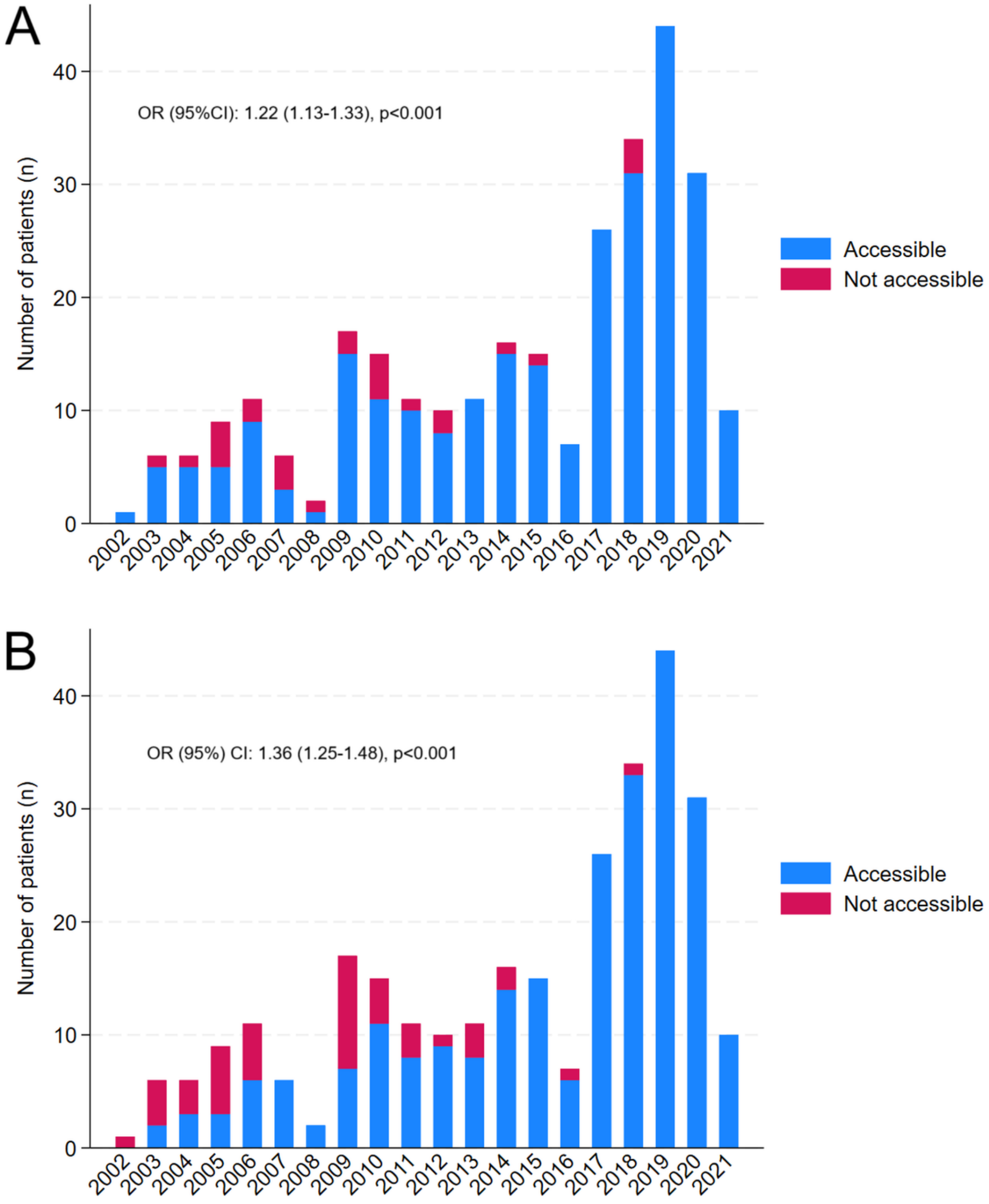

Supplementary Figure S4. Testing for tuberculosis prior to b/tsDMARD initiation

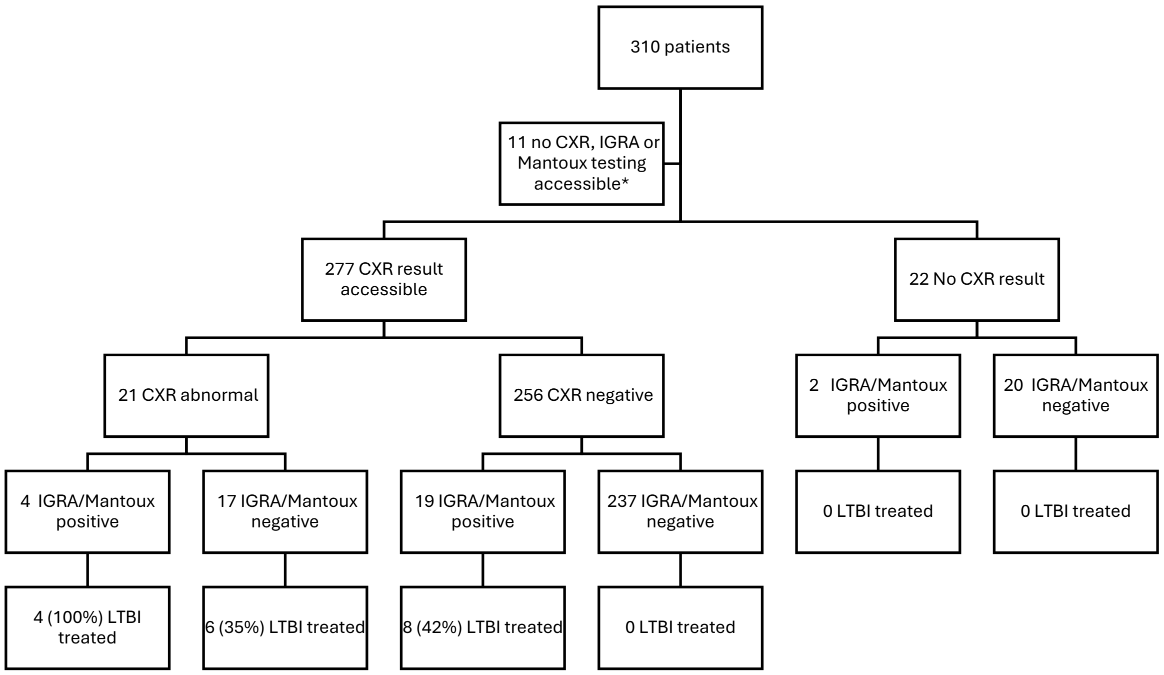

CXR: Chest x-ray
IGRA: Interferon gamma release assay
LTBI: latent tuberculosis infection
Mantoux positive: ≥5mm
*One of these patients was given chemoprophylaxis for latent TB in another centre prior to moving to Cairns

Supplementary Figure S5. Trends in testing for A. hepatitis B core antibody (HBcAb) and B. *Strongyloides stercoralis* serology prior to b/tsDMARD initiation

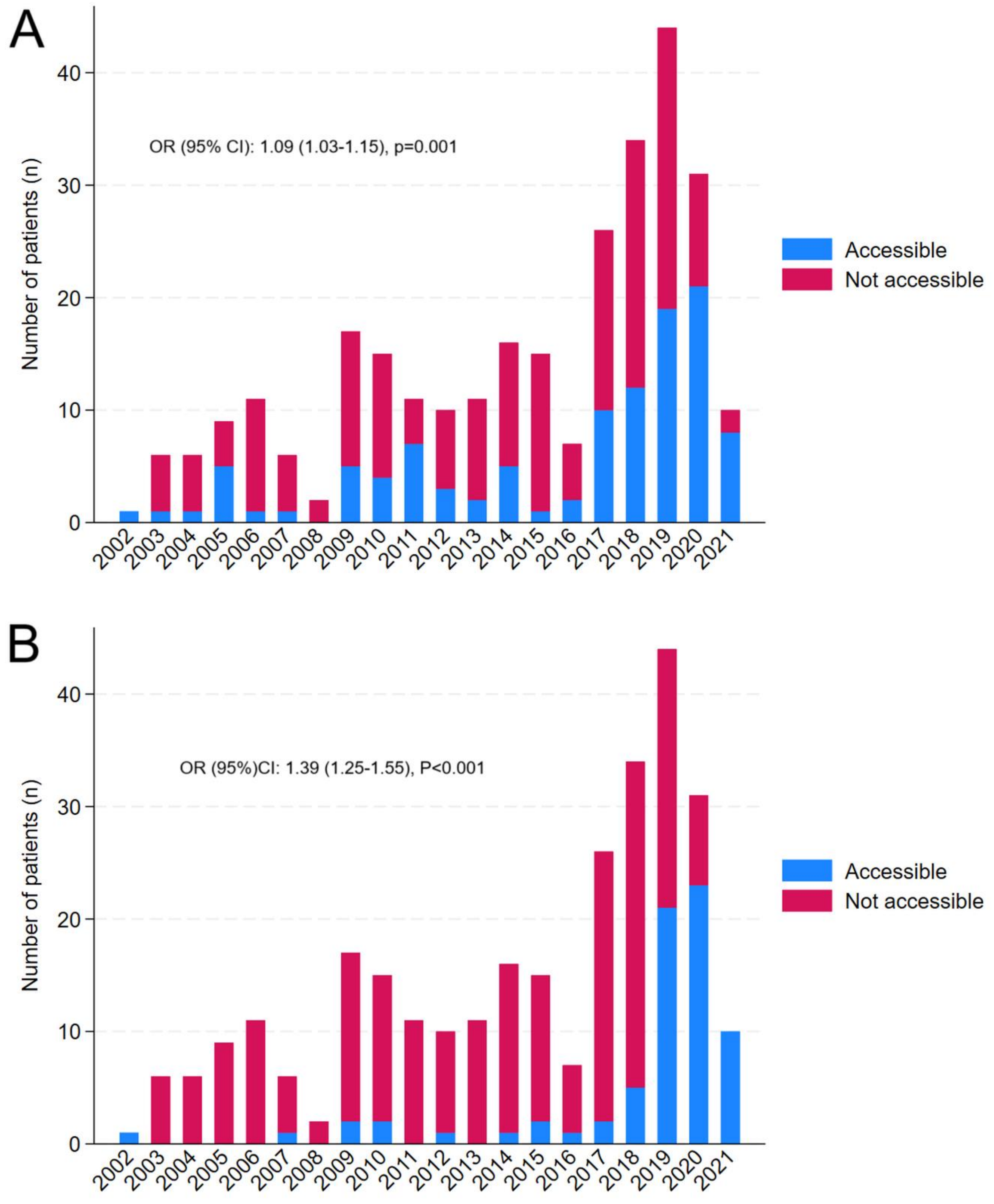

Supplementary Figure S6. Time to first serious infection between October 2012 and October 2021, stratified by whether patient received tumour necrosis factor inhibitor (TNFi) therapy during the study period.

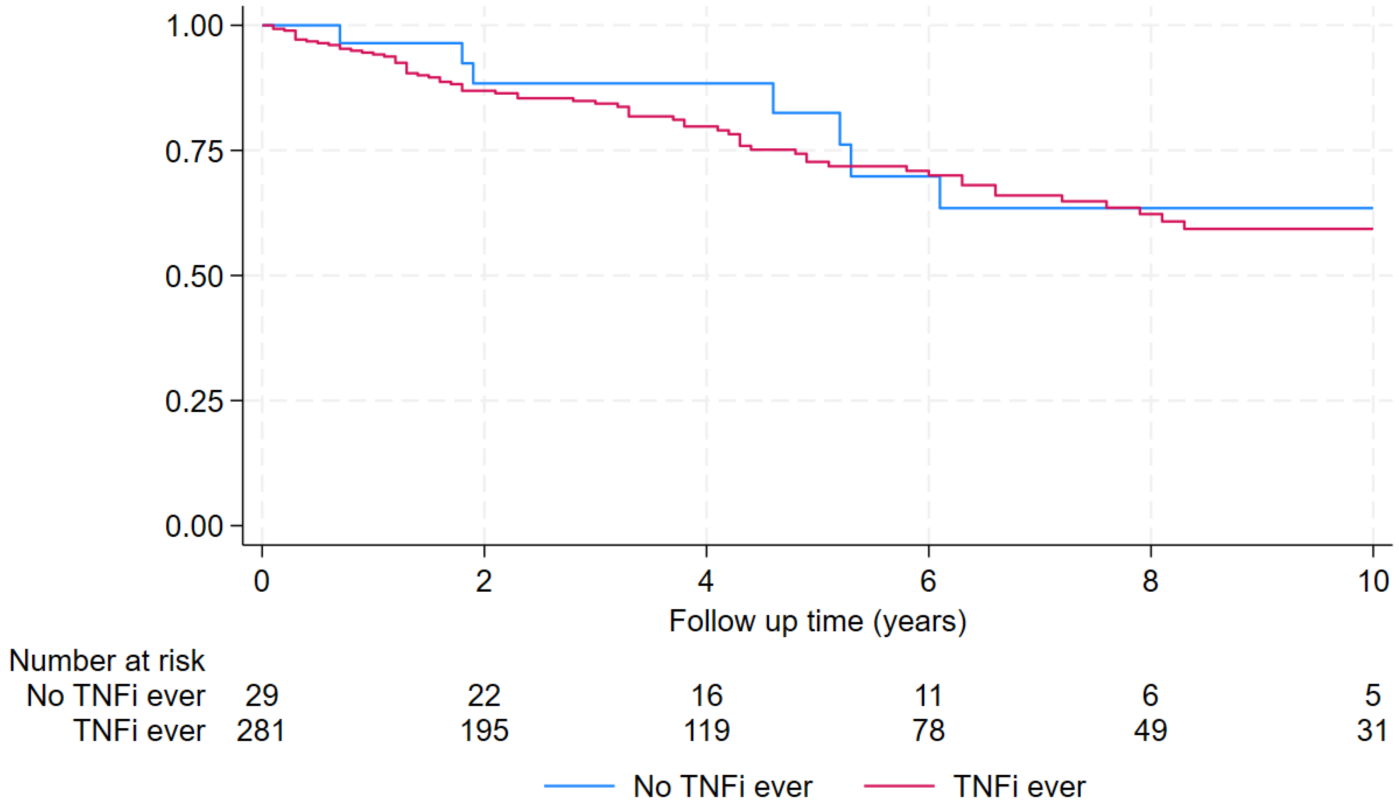

Supplementary Figure S7. Time to first serious infection between October 2012 and October 2021, stratified by whether patient received Janus kinase inhibitor (JAKi) therapy during the study period.

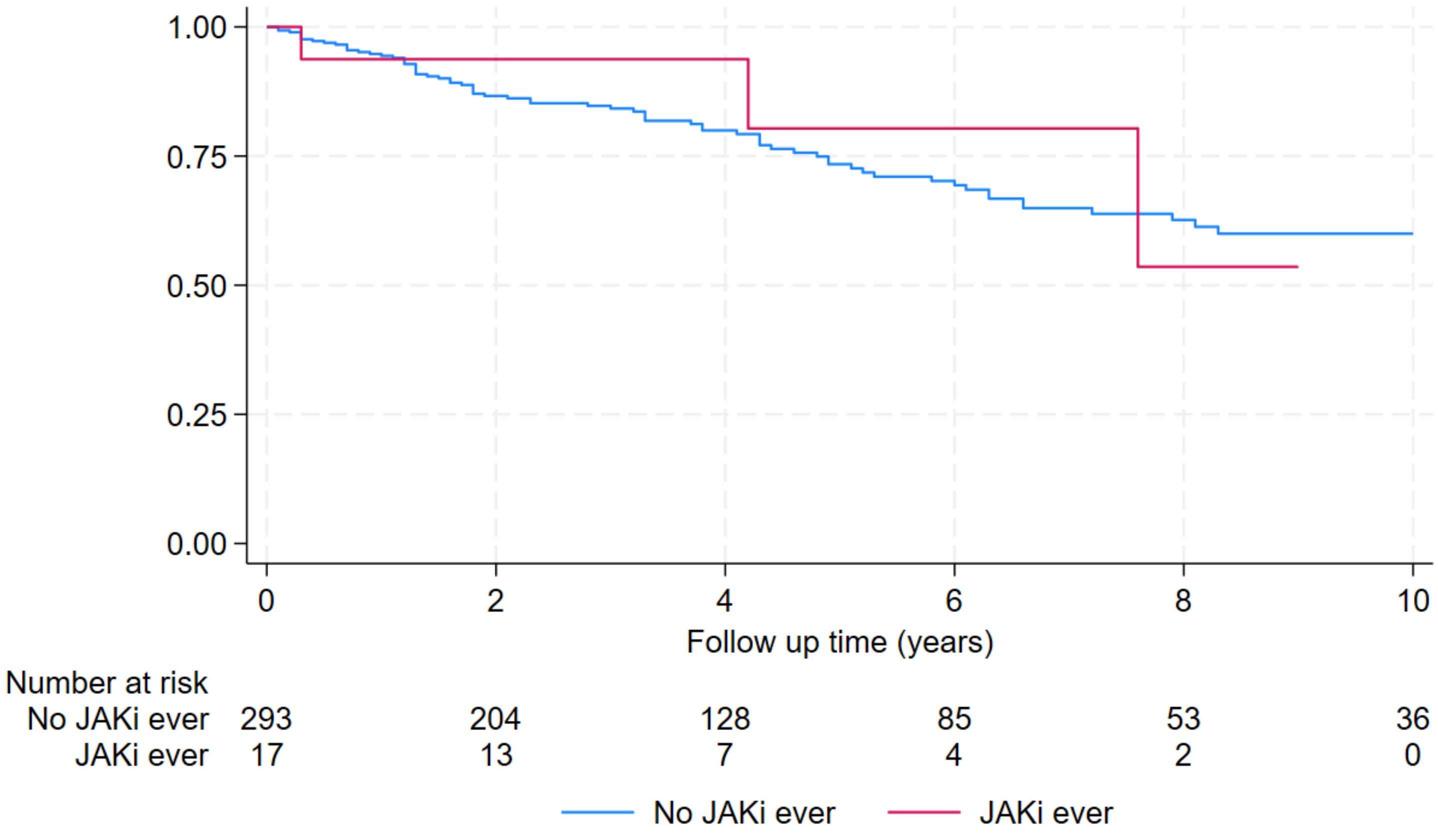

Supplementary Figure S8. Time to first serious infection between October 2012 and October 2021, stratified by whether patient received the B-cell depletion monoclonal antibody rituximab during the study period.

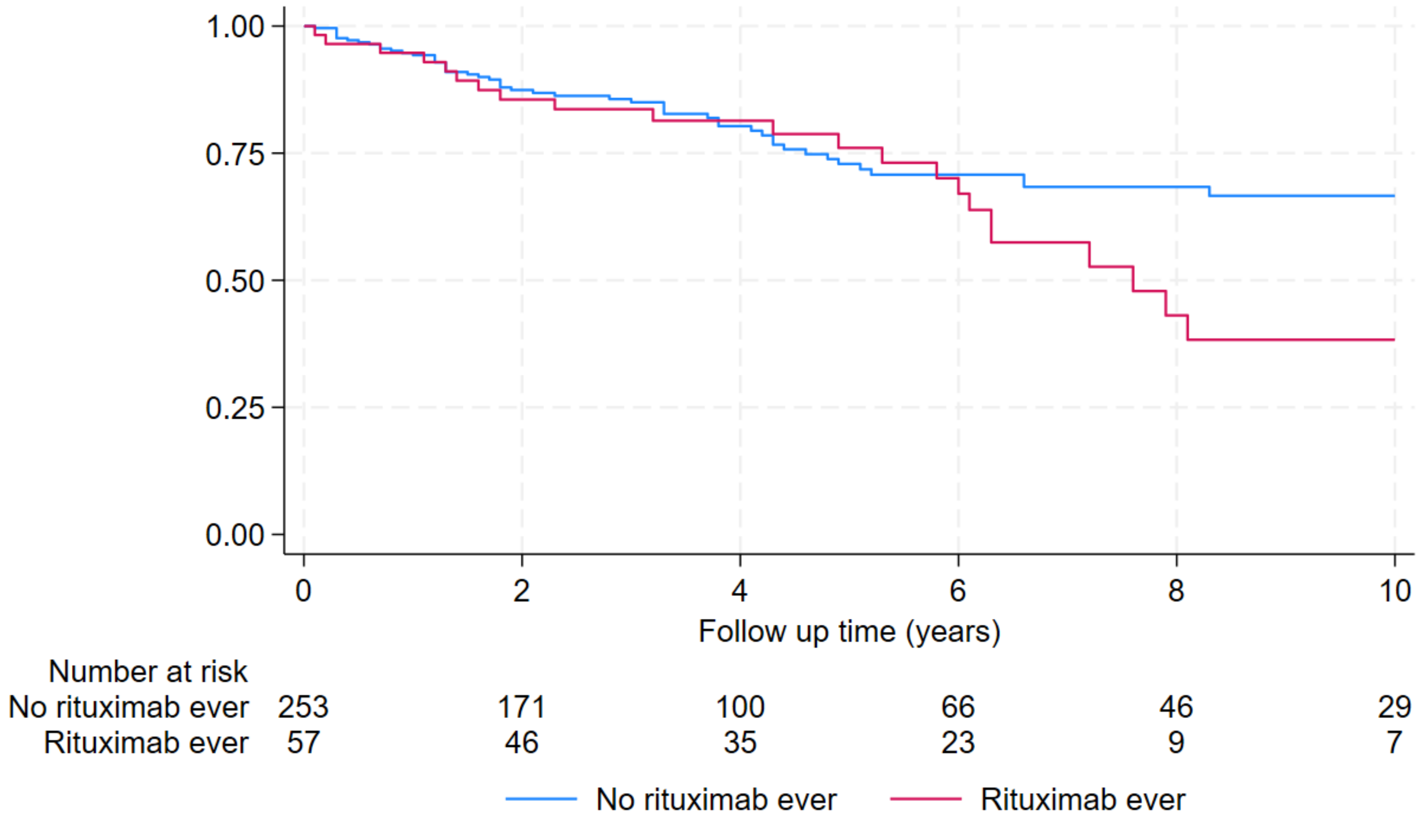

Supplementary Figure S9. Time to first serious infection between October 2012 and October 2021, stratified by whether the IL-6 inhibitor tocilizumab during the study period.

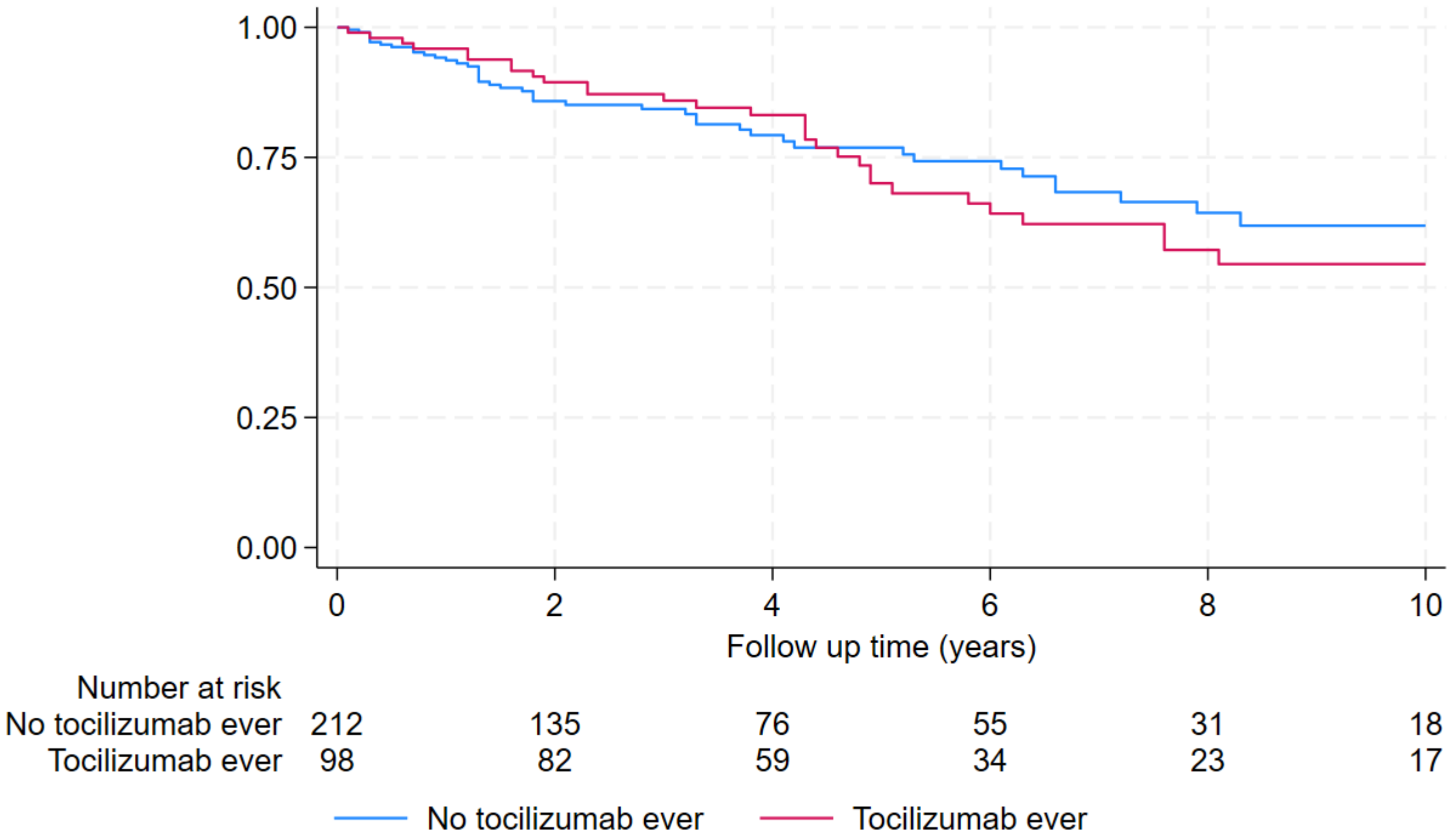

Supplementary Figure S10. Time to first serious infection between October 2012 and October 2021, stratified by whether patient received the CLTA4 inhibitor abatacept during the study period.

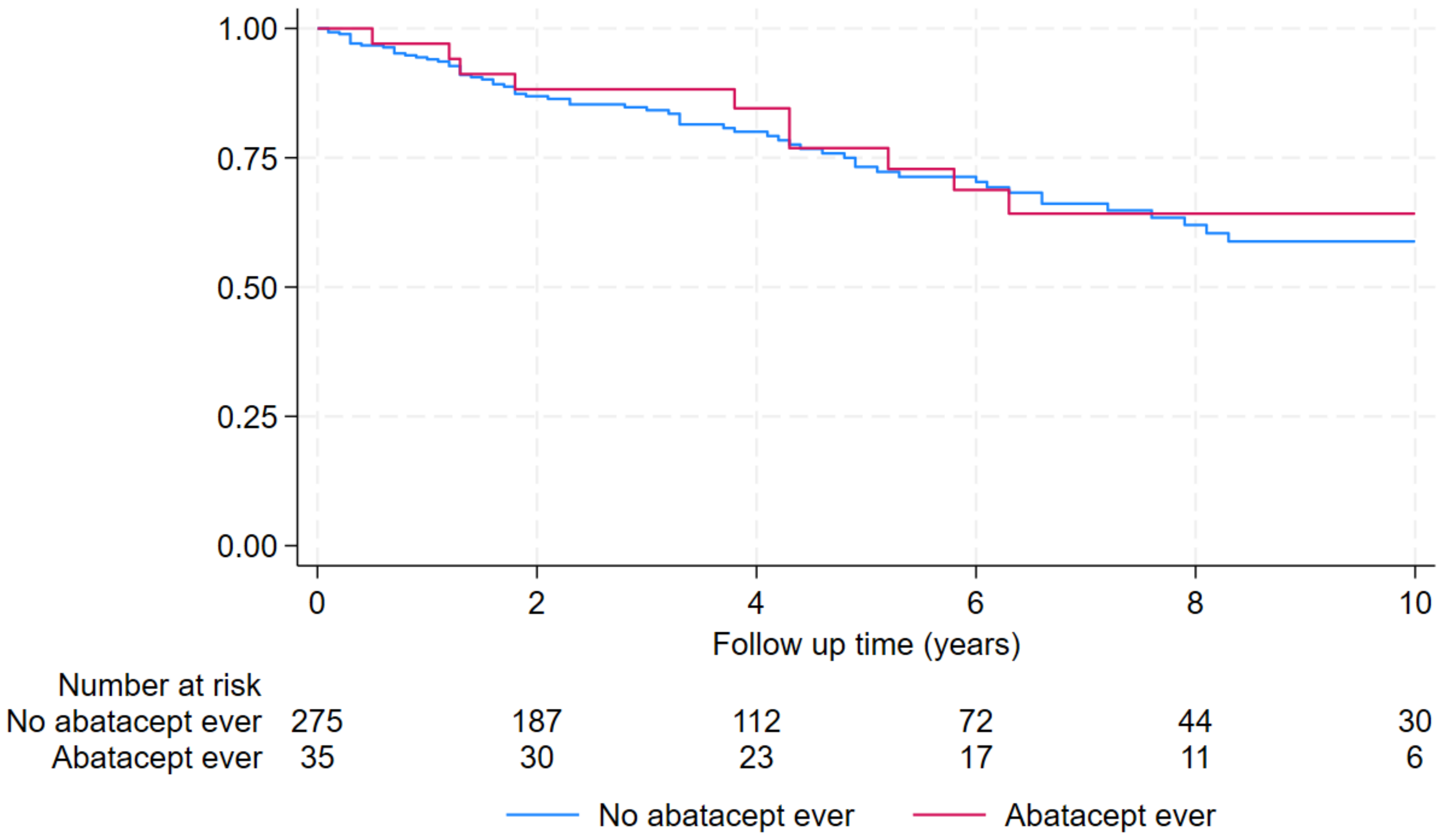

Supplement: Supplementary file 1 [file pathogens-13-00943-s001.zip › Supplementary table and figures for Pathogens 3255780R1.pdf]
